# Integrated transcriptomic and proteomic study on the different molecular mechanisms of PC12 cell growth on chitosan and collagen/chitosan films

**DOI:** 10.1093/rb/rbaa030

**Published:** 2020-08-31

**Authors:** Xiaoying Lü, Yan Huang, Yayun Qu, Yiwen Zhang, Zequn Zhang

**Affiliations:** 1 State Key Laboratory of Bioelectronics, School of Biological Science and Medical Engineering, Southeast University, Nanjing 210096, China; 2 SQ Medical Device Co., Ltd, Nanjing 210008, China

**Keywords:** chitosan film and collagen/chitosan film, protein adsorption and cell growth, cDNA microarray and proteomics, biological pathways

## Abstract

The purpose of this article is to integrate the transcriptomic analysis and the proteomic profiles and to reveal and compare the different molecular mechanisms of PC12 cell growth on the surface of chitosan films and collagen/chitosan films. First, the chitosan films and the collagen/chitosan films were prepared. Subsequently, the cell viability assay was performed; the cell viability of the PC12 cells cultured on the collagen/chitosan films for 24 h was significantly higher than that on the chitosan films. Then, with cDNA microarray, the numbers of differentially expressed genes of PC12 cells on the surface of chitosan and collagen/chitosan films were 13349 and 5165, respectively. Next, the biological pathway analysis indicated that the differentially expressed genes were involved in 40 pathways directly related to cell adhesion and growth. The integrated transcriptomic and our previous proteomic analysis revealed that three biological pathways—extracellular matrix–receptor interaction, focal adhesion and regulation of actin cytoskeleton—were regulated in the processes of protein adsorption, cell adhesion and growth. The adsorbed proteins on the material surfaces further influenced the expression of important downstream genes by regulating the expression of related receptor genes in these three pathways. In comparison, chitosan films had a strong inhibitory effect on PC12 cell adhesion and growth, resulting in the significantly lower cell viability on its surface; on the contrary, collagen/chitosan films were more conducive to promoting PC12 cell adhesion and growth, resulting in higher cell viability.

## Introduction

Chitosan has been extensively used in molecular imaging [1], gene therapy [2] and tissue engineering scaffold materials [3], on account of its favorable biocompatibility, biodegradability, physiological activity and penetration-enhancing property to improve the dissolution rate of poorly soluble drugs [4]. Yet its application is limited because of the poor solubility, low degradation rate and a certain inhibitory potency on cell adhesion and proliferation [5]. Collagen, as a main component of extracellular matrix (ECM), is involved in forming cytoskeleton and regulating cell adhesion, growth, proliferation and differentiation. Due to such excellent properties as biocompatibility, antioxidant activity and low immunogenicity, collagen has a wide range of biomedical applications in the field of tissue engineering, wound dressings and drug delivery [6]. However, it is difficult to use collagen alone because of its poor water resistance and susceptibility to degradation [7]. Therefore, some researchers tried using collagen/chitosan blends as an alternative for better biomedical purposes.

Mao *et al.* [8] tested and evaluated collagen/chitosan blends for acute systemic toxicity, subacute toxicity, intracutaneous irritation and skin sensitization in mice. The mice showed no poisoning, intracutaneous irritation or skin sensitization; and the visceral organs were normal. This indicated the excellent biocompatibility of collagen/chitosan blends. Gingras *et al*. [9] cultured human skin fibroblasts and keratinocytes on a collagen/chitosan sponge for 31 days to grow a reconstructed skin. The reconstructed skin was transplanted on the back of nude mice; linear arrangements of Schwann cells were observed 40 days after graft; nerve growth was first detected 60 days after graft and was more abundant along with these Schwann cell extensions later. This implied that the collagen/chitosan encouraged nerve growth. Zhan *et al*. [10] compared the growth of rabbit chondrocytes at passage 2 on collagen/chitosan porous scaffolds and on cell panels. On the surface of scaffolds, the proliferation of chondrocytes and the expressions of proteoglycan mRNA and type II collagen were significantly increased. This showed that collagen/chitosan was more conducive to promoting growth and proliferation of chondrocytes. The above studies at *in vivo*, cellular and molecular levels have demonstrated the good biocompatibility of collagen/chitosan blends. Collagen/chitosan blends has become a promising biomaterial in wound repair, artificial liver transplantation, peripheral nerve regeneration, skin tissue engineering, and more.

Protein adsorption to material surface is the first biological process after implantation of a biomaterial in the body, followed by cell adhesion. In order to understand ‘why’ collagen/chitosan films have better cellular compatibility than chitosan films, we previously underwent a proteomic study combining bioinformatic analysis on ‘material surface–protein adsorption–cell adhesion’, and compared the adsorbed proteins on the surface of chitosan films and collagen/chitosan films. The results revealed a difference in types and functions of adsorbed proteins on the surface of the two materials. Compared to chitosan films, collagen/chitosan surface supported stronger activation of ECM-receptor interaction pathway, focal adhesion pathway, regulation of actin cytoskeleton pathway and transforming growth factor-beta (TGF-β) signaling pathway in the PC12 cells. Moreover, collagen/chitosan surface had greater total adsorption of proteins containing Leu-Asp-Val (LDV) sequence and adsorbed more protein types containing Arg-Gly-Asp (RGD) sequence with greater individual adsorption. Therefore, collagen/chitosan films were more conducive to promoting cell adhesion and growth [11]. The fact that gene expression regulates cell behaviors drew us to hypothesize whether these adsorbed proteins further influence cell behaviors by affecting genes expression in cells.

To verify our hypothesis, a cell viability assay was performed for the PC12 cells cultured on the collagen and collagen/chitosan films for 24 h. Subsequently, cDNA microarray was used to analyze the intracellular gene expression profiles of the PC12 cells cultured on the chitosan films and the collagen/chitosan films; and was combined with the proteomic profiles of adsorbed proteins as we mentioned in our previous study [11] for a joint analysis. Furthermore, the different molecular mechanisms of PC12 cell behaviors on the surface of chitosan films and collagen/chitosan films were compared.

cDNA microarray has become an important method in the field of biocompatibility research [12] to analyze the influence on gene expression of cell–biomaterial interaction *in vitro*. It identifies differentially expressed genes, provides the essential gene information affecting biomaterial biocompatibility [13] and reveals the mechanism of cell–biomaterial interaction at the molecular level. However, no paper has reported any cDNA microarray study to explain the impacts of chitosan films and collagen/chitosan films on PC12 cell growth and proliferation.

Our previous study emphasized the functions of adsorbed serum proteins and the relevant biological pathways [11]. The joint analysis introduced in this article is novel and valuable because it revealed the essential biological signaling pathways relevant to cell adhesion and growth by integrating two processes together: (i) the adsorbed proteins on biomaterial surface influence genes expressions in cells, and (ii) important downstream genes in biological signaling pathways further regulate cell growth.

## Materials and methods

### Preparation of materials

The chitosan films and the collagen/chitosan films were prepared as described previously [11]. The ≥85% deacetylated chitosan powder (Jinan Heidebei Marine Bioengineering Co., Ltd., China) was dissolved in the 2% (v/v) acetic acid to formulate the 2% (w/v) chitosan solution. The chitosan films were formed on the glass slides via spin coating. After evaporation at 50°C for 12 h, the films were soaked in the 1% (w/v) NaOH solution for 24 h. Then the films were rinsed with ultrapure water three times and dried at room temperature. The 0.5 g acid-soluble type I collagen (Chengdu Xinji Bioactive Collagen Development Co., Ltd) was dissolved in 25 ml of 0.5 M acetic acid to formulate the 2% (w/v) collagen solution. This solution and the 2% chitosan solution were mixed with a volume ratio of 6:4. The collagen/chitosan films were fabricated on the glass slides via spin coating. After evaporation at 37°C for 12 h, the films were soaked in the 1% (w/v) NaOH solution for 24 h. Then the films were rinsed with ultrapure water three times and dried at room temperature. The chitosan and the collagen/chitosan films were sterilized for 1 h each side by UV lamp before biological experiments.

### Cell culture

PC12 cells were purchased from Shanghai Institutes for Biological Sciences. The cells were cultured in the DMEM high glucose medium (HyClone, USA) plus 10% fetal bovine serum (Hangzhou Sijiqing Bioengineering Co., China) and 1% (v/v) penicillin-streptomycin (HyClone, United States), and were incubated in a 37°C, 5% CO_2_ incubator with 100% relative humidity (Thermo Forma 3111, Thermo Fisher Scientific, USA). The experiments were performed with cells in logarithmic growth phase.

### Cell viability assay (Cell Counting Kit-8 assay, CCK8 assay)

The chitosan films and the collagen/chitosan films (8 mm × 8 mm) were placed in the 48-well plates as experimental groups. The PC12 cells were seeded on the material surface with a cell density of 1.875 × 10^4^ cells/cm^2^, and cultured for 4 h and 24 h, respectively. The negative control cells were cultured in the standard culture medium alone; the positive control cells were cultured in the medium plus 0.7% acrylamide solution without any biomaterial. At each time point, CCK8 solution was added to each well equal to 1/10 the media volume. Cells were incubated for another 3.5 h after adding CCK8, and then the absorbance at 450 nm was measured by a microplate reader (Biotek Synergy HT, USA). The experiment was replicated three times for each experimental and control group. The cell survival rate was calculated as follows:

$$\mathrm{Cell}\;\mathrm{survival}\;\mathrm{rate}\;(\%)=\frac{{\overset{-}{\mathrm{Absorbance}}}_{\mathrm{material}}}{{\overset{-}{\mathrm{Absorbance}}}_{\mathrm{negative}\;\mathrm{control}}}\times 100\%$$

### Proteomics experiment

The data of adsorbed serum proteins data on the chitosan films and the collagen/chitosan films were obtained from our previous study [11]. The two materials were incubated in the culture medium supplemented with 10% bovine serum at 37°C for 4 h. The numbers of serum proteins identified on the chitosan and the collagen/chitosan films were 104 and 98, respectively. The previous study emphasized the functions of adsorbed serum proteins and the relevant biological pathways. The present study further conducted a joint analysis of proteomics results with transcriptomic results, which provided new lines of inquiry to investigate an overall analysis of “material surface-protein adsorption-cell adhesion” relevant to cell adhesion and growth.

### cDNA microarray experiment

The PC12 cells were seeded with a cell density of 1.875 × 10^4^ cells/cm^2^ on the surface of chitosan films and collagen/chitosan films (52 mm in diameter), and on polystyrene petri dish (control). After 24-h incubation, the cells were lyzed using Trizol reagent and total RNA was extracted. The cDNA microarray (Rat GE 4 × 44K v3 Microarray) was performed by Shanghai Kangcheng Biological Engineering Co., Ltd. The experiment was replicated three times for each group.

The expression levels were calculated. The differentially expressed genes were screened out, for which the fold change cut-offs of >2 and <0.5 as criteria of up- and down-regulation, respectively (significance *P* values <0.01).

### Selection and verification of gene candidates for quantitative real-time PCR (qRT-PCR)

#### Selection criteria

Quantitative RT-PCR is a commonly used validation tool for verifying the reliability of the cDNA microarray data. The candidate genes selected for qRT-PCR were required to match to all the following criteria: (i) higher differential expression; (ii) involvement in biological signaling pathways of cell adhesion and growth as described in the reference; (iii) involvement in focal adhesion and regulation of actin cytoskeletal; and (iv) greater fluorescence in cDNA microarray [14–18].

#### qRT-PCR

Four genes (Pak1, Actn1, Bcar1 and Aktl) were selected for validation by qRT-PCR. Calculations were performed using the 2^−ΔΔCt^ method; all C_t_ values were normalized to the endogenous control GAPDH. The qRT-PCR was conducted by Shanghai Kangcheng Biological Engineering Co., Ltd.

### Biological pathway analysis of differentially expressed genes

The biological pathway analysis of differentially expressed genes was performed by DAVID (http://david.abcc.ncifcrf.gov/).

### Joint biological pathway analysis of differentially expressed genes and adsorbed proteins

The biological pathways involving differentially expressed genes were compared with the pathways contributed by adsorbed serum proteins as we discussed in our previous paper [11], to further investigate the different molecular mechanisms of PC12 cell adhesion and growth affected by chitosan films and collagen/chitosan films.

### Statistical analysis

All experimental data were expressed as mean ± standard deviation. Student’s *t*-test was performed unless otherwise noted. *P *<* *0.05 was considered to indicate significant difference; *P *<* *0.01 was considered to indicate a highly significant difference. All experiments were repeated at least three times.

## Results and discussion

### Results of cell viability (CCK8 assay)

**Figure 1.**
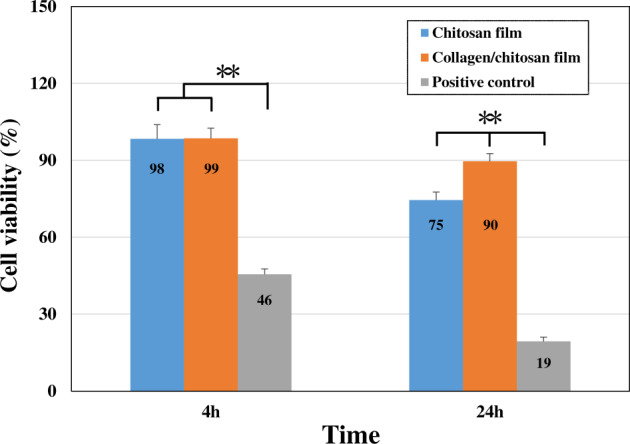
The percent cell viability of PC12 cells cultured for 4 and 24 h. (**: *P* < 0.01)

The cell viability data were presented in Fig. 1. After 4 and 24 h, the percent cell viability of both experimental groups was significantly higher than that of positive control group (*P* < 0.01). At 4 h, there was no significant difference in the percent cell viability between the PC12 cells cultured on the surface of chitosan films and collagen/chitosan films (98.4% and 98.6%, respectively). At 24 h, the percent cell viability for cells on the surface of collagen/chitosan films was 89.60%, significantly higher than that, 74.50%, on the chitosan films (*P* < 0.01). This result agreed fairly well with our previous observation using MTT assay [11] that the collagen/chitosan films promoted cell growth more significantly.

### Results of cDNA microarray

The differentially expressed genes in the PC12 cells cultured for 24 h were screened out, the number of which were summarized in Table 1. The cells cultured on the chitosan films had a larger number of differentially expressed genes indicating greater change of gene expression profiling compared to the cells cultured on the collagen/chitosan films, and had more up-regulated genes than down-regulated genes; whereas the cells cultured on the collagen/chitosan films had more down-regulated than up-regulated genes.

**Table 1.** Number of differentially expressed genes in the PC12 cells cultured on the chitosan films and the collagen/chitosan films for 24 h

Materials	Number of up-regulated genes	Number of down-regulated genes	Total number of differentially expressed genes
Chitosan film	7076	6273	13 349
Collagen/chitosan film	2473	2692	5165

It has been demonstrated that such physical and chemical cues of biomaterials as surface morphology [13], different degree of charges [19] or matrix stiffness [20, 21], influence gene responses, protein adsorption and cell behaviors. Our previous research indicated that the difference in surface functional groups characterized the difference in hydrophilicity and hydrophobicity between the chitosan films and the collagen/chitosan films. This determined the different types and functions of adsorbed proteins [11], resulting in different gene expression profiles of PC12 cells cultured on the chitosan and the collagen/chitosan films.

### Results of quantitative real-time PCR (qRT-PCR)

**Figure 2.**
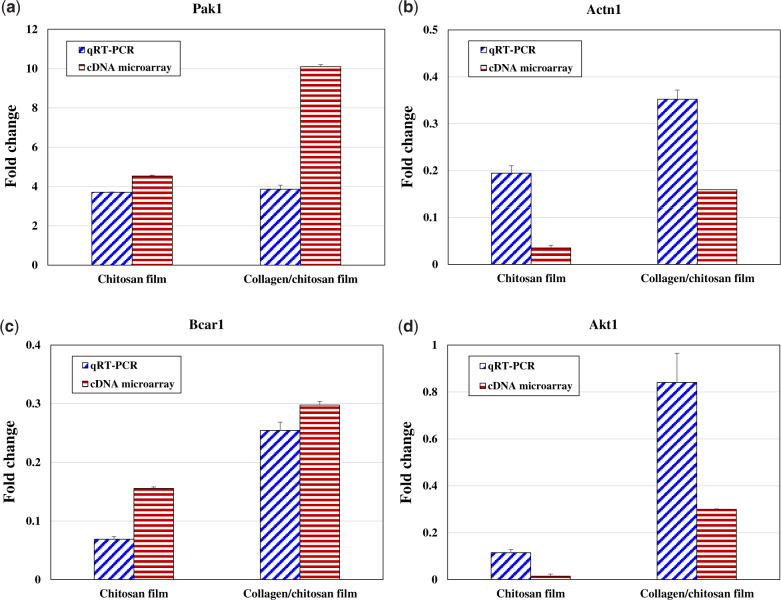
Comparison of the expression levels [(**a**) Pak1, (**b**) Actn1, (**c**) Bcar1 and (**d**) Akt1] in the qRT-PCR and the cDNA microarray experiments

The expression of four genes (Pak1, Actn1, Bcar1, Aktl) in the qRT-PCR was compared to their expression in the cDNA microarray (Fig. 2). The reliability of cDNA microarray was verified according to the same direction of change in expression for these four genes in the RT-PCR and in the cDNA microarray. Pak1 was up-regulated; Actn1, Bcar1 and Akt1 were down-regulated.

Considering the principles, inherent pitfalls and vastly different normalization procedures between two measurements [22], it was acceptable that the fold change results determined by qRT-PCR were not closely correlated to the fold change assessed for the same genes by cDNA microarray. The qRT-PCR allows for the direct detection of fluorescent signals produced and monitored during the amplification process in the liquid-phase system [23]. The cDNA microarray data are obtained through the acquisition of fluorescent hybridization signals using solid-phase interface probes. Variability in biological procedures, which cannot be controlled, enables a great impact on both measurements. What’s more, some of the inherent pitfalls cannot be eliminated in the light of quantitative measurements. Though the variation of correlation between two measurements made the interpretation gene-specific, the same direction of increase or decrease in expression was observed for each of these four genes.

### Biological pathway analysis of differentially expressed genes

DAVID was used to determine the biological signaling pathways involving differentially expressed genes in the PC12 cells cultured for 24 h. 89 pathways were significantly affected by the genes in the chitosan group, whereas 86 pathways in the collagen/chitosan group. Among these, 40 pathways were directly engaged in cell adhesion and growth in each group (Supplementary Table 1).

### Joint biological pathway analysis of differentially expressed genes and adsorbed proteins

In this article, the abovementioned pathways were further compared with the nine pathways contributed by adsorbed proteins as ligand-membrane receptors in our previous proteomic study [11]. In the chitosan group, adsorbed serum proteins and differentially expressed genes were both involved in three pathways (Table 2, 1st–3rd); differentially expressed genes alone were involved in 1 pathway (4th); adsorbed proteins alone were involved in three pathways (6th–8th). In the collagen/chitosan group, adsorbed proteins and differentially expressed genes were both involved in five pathways (1st–5th); adsorbed proteins alone were involved in four pathways (6th–9th). Of them, four pathways (1st–3rd, 9th) were directly engaged in cell adhesion, growth and proliferation. The first three pathways were chosen to be emphasized in this article because they played very important roles from the perspectives of both differentially expressed genes and adsorbed proteins.

**Table 2.** Number of the differentially expressed genes and the adsorbed proteins involved in the nine biological pathways in the chitosan and the collagen/chitosan groups

No.	Biological pathways	Number of differentially expressed genes		Number of adsorbed proteins [13]	
		Chitosan film	Collagen/chitosan film	Chitosan film	Collagen/chitosan film
**1**	**Focal adhesion**	**126**	**72**	**2**	**4**
**2**	**Regulation of actin cytoskeleton**	**118**	**64**	**2**	**2**
**3**	**ECM–receptor interaction**	**51**	**29**	**2**	**4**
4	Chemokine signaling	95	53	0	1
5	Cytokine–cytokine receptor interaction	—	52	0	1
6	Complement and coagulation cascades	—	—	20	17
7	Neuroactive ligand–receptor interaction	—	—	2	2
8	Adipocytokine signaling pathway	—	—	1	1
9	TGF-β signaling pathway	—	—	0	1

The ECM-receptor interaction pathway will be discussed first since most of adsorbed proteins are ECM proteins that initially mediate cell adhesion to a biomaterial.

#### ECM–receptor interaction pathway

ECM, composed of macromolecules, plays a prominent role in morphogenesis and in providing structural and functional support for tissues and organs. The specific interaction between ECM and cells is mediated by transmembrane proteins including integrins, proteoglycans and other cell-surface components; and directly or indirectly regulates cellular processes such as cell adhesion, migration, differentiation, proliferation and apoptosis.

**Figure 3.**
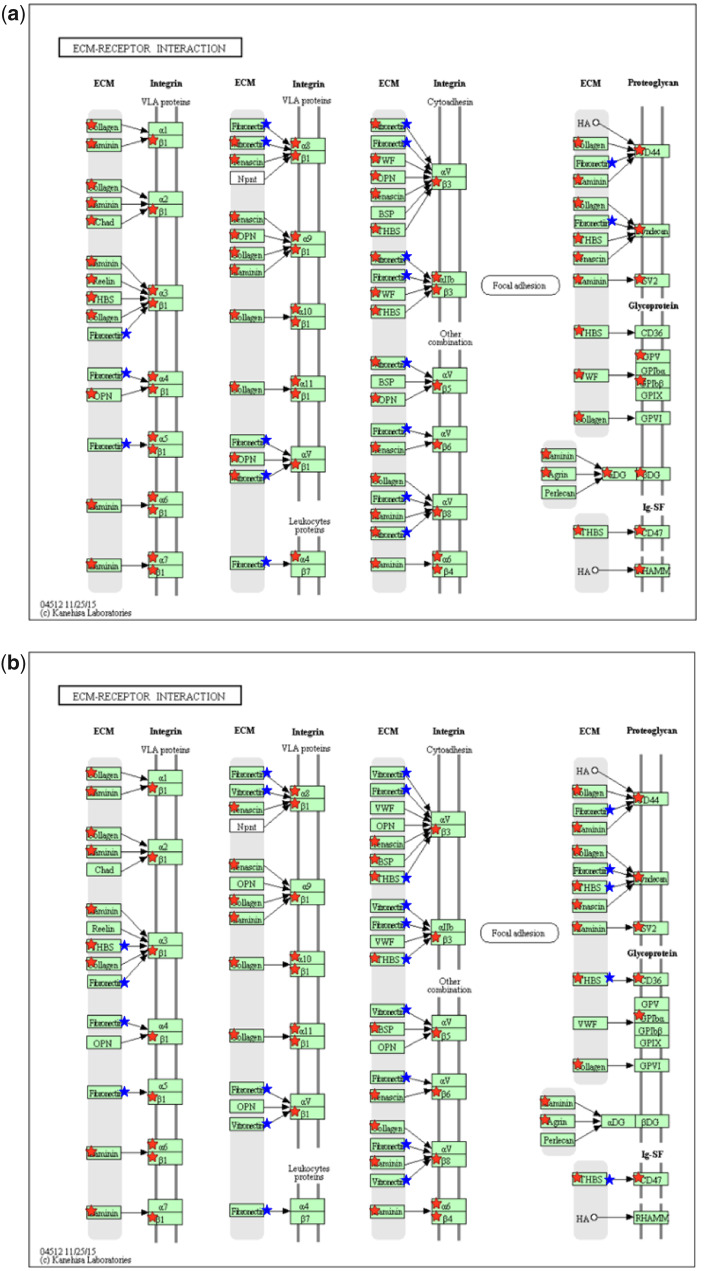
The differentially expressed genes and the adsorbed serum proteins involved in the ECM–receptor interaction pathway [24] in the PC12 cells cultured on (**a**) the chitosan films and (**b**) the collagen/chitosan films for 24 h. The red star represents a differentially expressed gene; the blue star represents an adsorbed protein

Figure 3 illustrated the differentially expressed genes (red ⋆) and the adsorbed serum proteins (blue ⋆) [11] involved in the ECM–receptor interaction pathway in the PC12 cells cultured on two materials for 24 h. The adsorbed proteins on the chitosan films were vitronectin (VTN) and fibronectin 1 (FN1); the adsorbed proteins on the collagen/chitosan films were VTN, FN1, thrombospondin 1 (THBS1) and thrombospondin 4 (THBS4). These adsorbed proteins interact with four types of receptors: integrins, proteoglycans, glycoproteins and immunoglobulin (Ig) superfamily (Fig. 3). Table 3 listed the differentially expressed genes that encoded the receptors binding to adsorbed proteins: 12 genes (6 up-regulated and 6 down-regulated) in the chitosan group, whereas 7 genes (5 up-regulated and 2 down-regulated) in the collagen/chitosan group. Among them, eight genes were differentially expressed in the chitosan group alone, whereas three genes were differentially expressed in the collagen/chitosan group alone. Among the four genes that were differentially expressed in both groups, their expression levels in the chitosan group were higher than those in the collagen/chitosan group.

**Table 3.** The differentially expressed genes that encoded the receptors binding to adsorbed proteins involved in the ECM-receptor interaction pathway

Types of genes	Gene symbol	Chitosan film	Collagen/chitosan film
Integrin genes	Itga2b	3.274 ± 0.228↑	
	Itga3	0.183 ± 0.000↓	
	Itga4	0.178 ± 0.000↓	
	Itga5	0.074 ± 0.019↓	
	Itga6		0.257 ± 0.007↓
	Itga8	4.594 ± 0.010↑	3.558 ± 0.013↑
	Itgb1	0.011 ± 0.000↓	0.199 ± 0.001↓**
	Itgb5	0.021 ± 0.014↓	
	Itgb6	2.094 ± 0.054↑	
	Itgb8		2.024 ± 0.037↑
Proteoglycan-encoding genes	Cd44	2.234 ± 0.550↑	
	Sdc1	2.046 ± 0.010↑	
	Sdc4	6.220 ± 1.867↑	3.065 ± 0.010↑**
Glycoprotein-encoding genes	RGD1565355		7.887 ± 0.077↑
Immunoglobulin genes	Cd47	0.031 ± 0.000↓	2.156 ± 0.030↑**
Number of up-regulated genes^a^		6	5
Number of down-regulated genes^b^		6	2

^a^ Up-regulated genes (expression value > 2).

^b^ Down-regulated genes (expression value < 0.5).

** *P* < 0.01, fold change of one gene between chitosan and collagen/chitosan groups was considered to have a very significant difference.

#### Focal adhesion pathway

Focal adhesions are the clusters of integrin transmembrane receptors that mechanically couple ECM to actin cytoskeleton [25]. Focal adhesions sense and respond to variations in force transmission along a chain of protein–protein interactions linking successively actin filaments, actin-binding proteins, integrins and ECM to adapt cell-matrix adhesion to the composition and mechanical properties of the ECM. Some components of focal adhesions serve as structural connection between membrane receptors and actin cytoskeleton; others, including adaptor proteins, kinases and substrates, phosphatases and substrates [26], serve as important signaling components in cell adhesion and proliferation.

**Figure 4.**
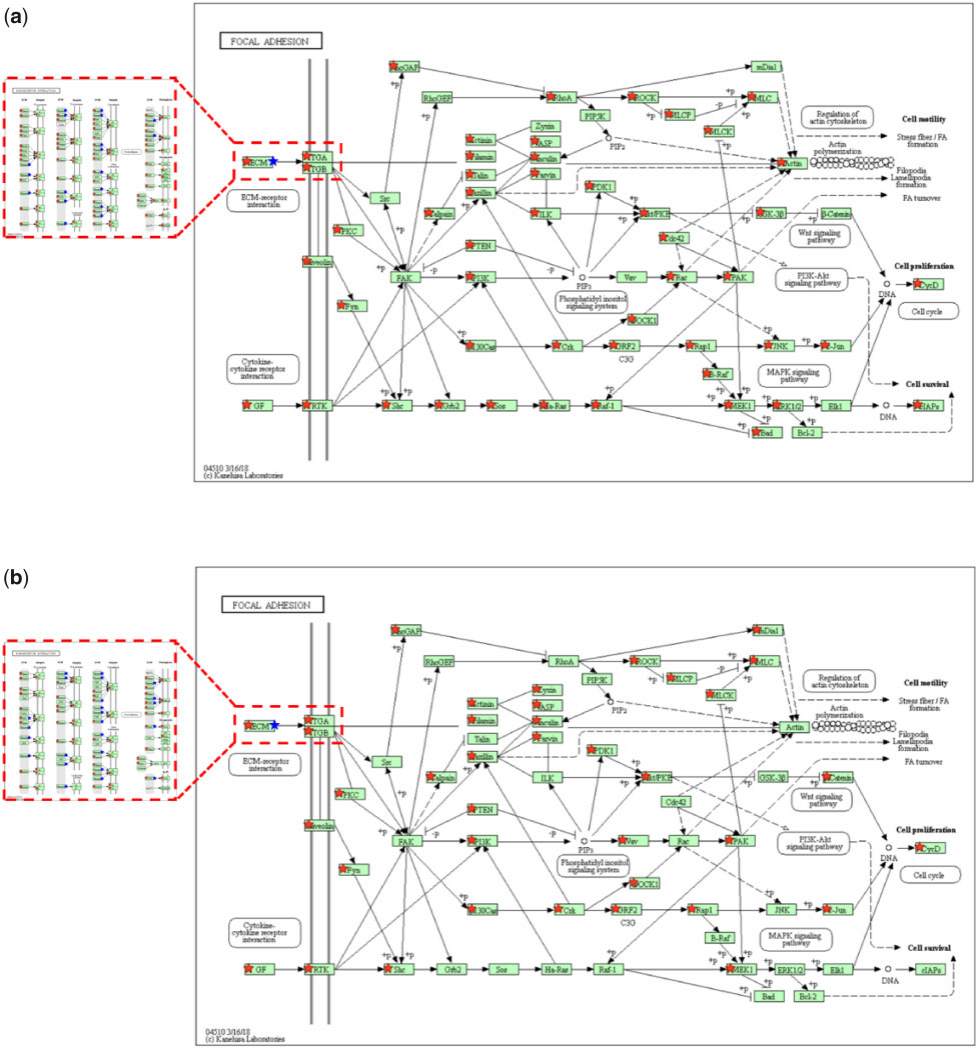
The differentially expressed genes and adsorbed serum proteins involved in the focal adhesion pathway [24] in the PC12 cells cultured on (**a**) the chitosan films and (**b**) the collagen/chitosan films for 24 h. The red star represents a differentially expressed gene; the blue star represents an adsorbed protein

Figure 4 illustrated the differentially expressed genes (red ⋆) involved in the focal adhesion pathway in the PC12 cells cultured on two materials for 24 h. The aforementioned upstream ECM–receptor interaction pathway and the adsorbed serum proteins involved (blue ⋆) [11] were represented in the red dashed boxes. The adsorbed proteins were VTN, FN1, THBS1 and THBS4. The adsorbed proteins interact with integrin α subunits (ITGA) and integrin β subunits (ITGB). In the chitosan group, two adsorbed proteins (VTN, FN1) bound to five ITGA genes (Itga2b↑, Itga3↓, Itga4↓, Itga5↓, Itga8↑) and 3 ITGB genes (Itgb1↓, Itgb5↓, Itgb6↑). In the collagen/chitosan group, four proteins (VTN, FN1, THBS1, THBS4) bound to two ITGA genes (Itga6↓, Itga8↑) and two ITGB genes (Itgb1↓, Itgb8↑) (Table 3).

Table 4 listed the number of differentially expressed ITGA and ITGB genes that encoded the integrins binding to the adsorbed proteins in the focal adhesion pathway: in the chitosan group were five ITGA genes (2 up-regulated, 3 down-regulated) and three ITGB genes (1 up-regulated, 2 down-regulated); whereas in the collagen/chitosan group were two ITGA genes (1 up-regulated, 1 down-regulated) and two ITGB genes (1 up-regulated, 1 down-regulated). There were more down-regulated than up-regulated ITGA and ITGB genes in the chitosan group.

**Table 4.** The number of differentially expressed ITGA and ITGB genes involved in the focal adhesion pathway in the experimental groups

Kinds of genes	Materials	Number of up-regulated genes	Number of down-regulated genes
ITGA coding genes	Chitosan film	2	3
	Collagen/chitosan film	1	1
ITGB coding genes	Chitosan film	1	2
	Collagen/chitosan film	1	1

Integrins are cell-surface transmembrane glycoproteins and are widely distributed on several cell types. They function as principal bi-directional cell surface linkers between ECM and intracellular actin cytoskeleton. Integrins can signal through cell membrane and regulate various cellular activities. They phosphorylate focal adhesion kinase and other tyrosine kinases, modify the organization of actin cytoskeleton, trigger ion fluxes and thus activate multiple signaling pathways including regulation of actin cytoskeleton, focal adhesion and mitogen-activated protein kinase (MAPK). And many integrin signals converge on cell cycle regulation, directing cells to live or die, to migrate, to proliferate or to exit the cell cycle and differentiate [27–30].

Four important downstream integrin genes—Actn1, Vcl, Flna and Ilk—in the focal adhesion pathway [31] were down-regulated in the chitosan group; whereas Actn1 and Flna were down-regulated yet Vcl was up-regulated in the collagen/chitosan group (Table 5). Compared to the collagen/chitosan group, the chitosan group had a larger number of down-regulated genes and lower expression of three differentially expressed genes—Actn1, Vcl and Flna. Actinin alpha 1 (ACTN1), vinculin (VCL) and filamin A (FLNA) are cytoplasmic adaptor proteins associated with protein–protein interactions in signal transduction. ACTN1 is involved in cell cycle [32]. VCL not only contributes to cell adhesion, cell spreading and stability and robustness of focal adhesion but also plays an important role in the regulation of cell migration and anti-apoptotic signaling pathways [33–35]. FLNA is an actin-binding protein that induces high-angle cross-linking of actin filaments. Cells lacking FLNA expression have a reduced cytoplasmic viscoelasticity, unstable surface and are unable to undergo translational locomotion [36]. Integrin-linked kinase (ILK) is a serine-threonine protein kinase located in focal adhesions and regulates cell growth, proliferation, differentiation, migration and invasion [37]. In many cancers, overexpression of ILK leads to increased cancer growth and spread by promoting cell proliferation, migration and epithelial-mesenchymal transition [38]. As a key transducer in the focal adhesion pathway [31], the down-regulation of Ilk in the chitosan group might lead to malfunction of focal adhesion and then affect the mechanical connection and signal transduction between the cell and its ECM.

**Table 5.** Gene expression of important downstream integrin genes in the focal adhesion pathway (red ⋆)

Gene symbol	Chitosan film	Collagen/chitosan film
Actn1	0.035 ± 0.006↓	0.159 ± 0.001↓**
Vcl	0.091 ± 0.000↓	2.437 ± 0.010↑**
Flna	0.001 ± 0.000↓	0.072 ± 0.012↓**
Ilk	0.144 ± 0.020↓	

** *P* < 0.01, fold change of one gene between chitosan and collagen/chitosan groups was considered to have a very significant difference.

Overall, the adsorbed proteins on two material surfaces influenced the focal adhesion pathway by regulating the expression of integrin genes in cells. The chitosan films rather than the collagen/chitosan films were more inhibitory to PC12 cell adhesion and growth.

#### Regulation of actin cytoskeleton pathway

Cytoskeleton as an important network of protein fibers in eukaryotic cells maintains cellular morphology, provides the correct positioning of organelles, facilitates cell movement, supports cell differentiation and division and aids in energy conversion, signaling transduction material transfer, etc. In a general sense, cytoskeleton includes cytoplasmic skeleton (microfilaments, microtubules and intermediate filaments), cell membrane skeleton, nuclear skeleton and ECM, which are structurally connected to each other and run through the dynamic network of the nucleus and cytoplasm [39]. Actin is a critical component of cytoskeletal microfilaments and can be present in two forms as either a monomeric globule called G-actin or a linear polymeric microfilament called F-actin. G-actins polymerize to form F-actin. F-actin is a component of stress fibers and microfilament network. Cells can respond to extracellular stimuli through remodeling of the actin cytoskeleton [40, 41].

**Figure 5.**
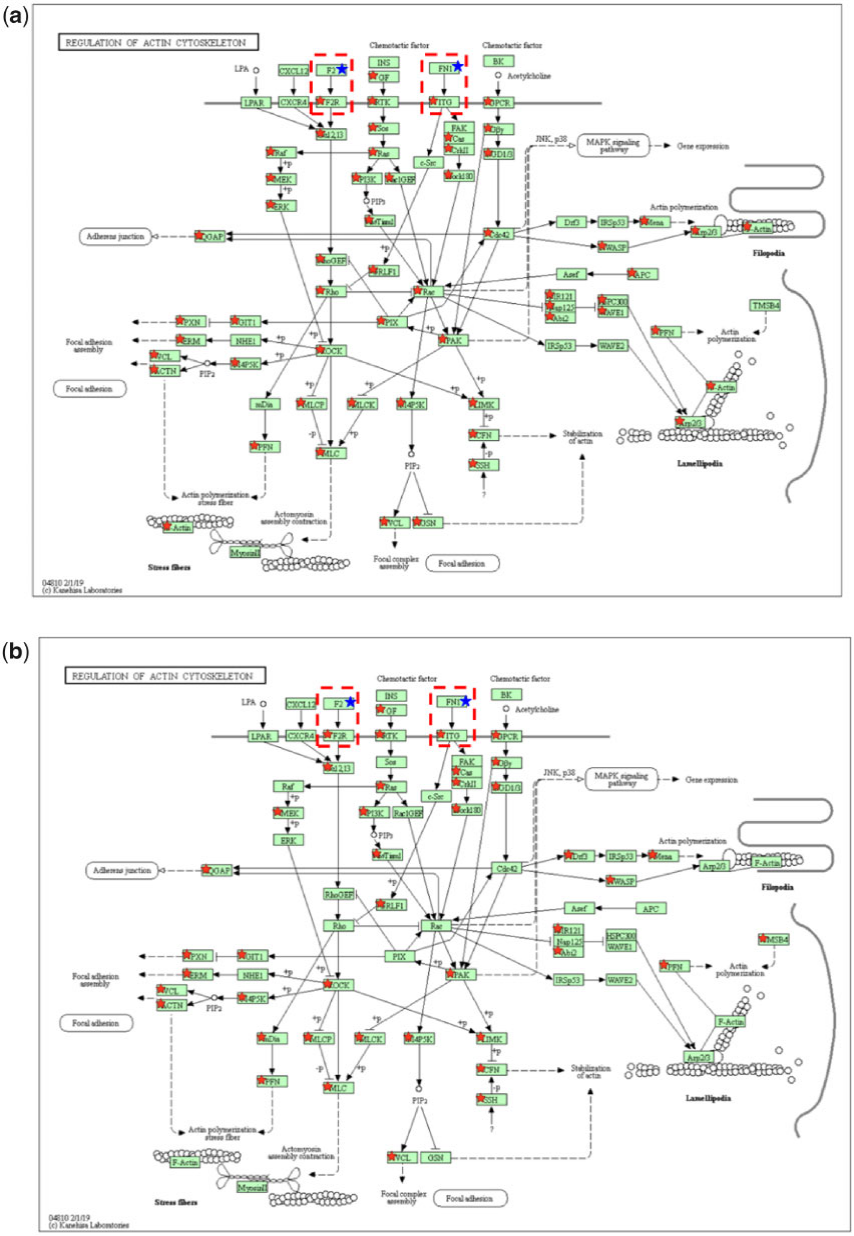
The differentially expressed genes and adsorbed serum proteins involved in the regulation of actin cytoskeleton pathway [24] in the PC12 cells cultured on (**a**) the chitosan films and (**b**) the collagen/chitosan films for 24 h. The red star represents a differentially expressed gene; the blue star represents an adsorbed protein

Figure 5 illustrated the differentially expressed genes (red ⋆) and the adsorbed serum proteins (F2 and FN1, blue ⋆) [11] involved in the regulation of actin cytoskeleton pathway in the PC12 cells cultured on two materials for 24 h. The coagulation factor II (F2) bound to the cell surface receptor F2R directly; FN1 interacted with integrins directly (as shown in the red dashed boxes). The expression levels of 15 integrin genes and one F2R gene in the chitosan group were compared to those of 10 integrin genes and one F2R gene in the collagen/chitosan group (Fig. 5 and Table 6).

**Table 6.** The differentially expressed genes that encoded the receptors binding to F2 and FN1 involved in the regulation of actin cytoskeleton pathway

Types of genes	Gene symbol	Chitosan film	Collagen/chitosan film
F2R gene binding to F2	F2r	0.162↓	0.205↓
Integrin genes binding to FN1	Itga2b	3.274 ± 0.228↑	
	Itga3	0.183 ± 0.000↓	
	Itga4	0.178 ± 0.000↓	
	Itga5	0.074 ± 0.019↓	
	Itga6	0.125 ± 0.000↓	0.257 ± 0.007↓**
	Itga7	0.013 ± 0.000↓	
	Itga8	4.594 ± 0.010↑	3.558 ± 0.013↑**
	Itga9	3.383 ± 1.537↑	
	Itga10	10.033 ± 1.446↑	7.789 ± 0.114↑**
	Itga11	6.939 ± 0.497↑	2.153 ± 0.040↑**
	Itgad	3.231 ± 0.050↑	
	Itgam	6.303 ± 0.203↑	2.355 ± 0.046↑**
	Itgax		3.073 ± 0.073↑
	Itgb1	0.011 ± 0.000↓	0.199 ± 0.001↓**
	Itgb2		4.428 ± 0.060↑
	Itgb4		2.955 ± 0.044↑
	Itgb5	0.021 ± 0.014↓	
	Itgb6	2.094 ± 0.054↑	
	Itgb8		2.024 ± 0.037↑

** *P* < 0.01, fold change of one gene between chitosan and collagen/chitosan groups was considered to have a very significant difference.

F2 binding to F2R transmits extracellular signals into intracellular signals, activates RhoA GTPase, induces assembly of F-actin cytoskeleton and thus promotes cell migratory behavior [42]. F2r was down-regulated in both chitosan and collagen/chitosan groups with a lower expression level (greater down-regulation) in the chitosan group (Table 6), indicating a stronger inhibitory effect of the chitosan films on generating and assembling F-actin stress fibers. In addition, among 15 integrin genes binding to FN1 in the chitosan group, 8 genes were up-regulated yet 7 genes were down-regulated. In the collagen/chitosan group, 8 of the 10 integrin genes were up-regulated yet 2 genes were down-regulated. In general, the up-regulated genes played the predominant role in the regulation of actin cytoskeleton pathway.

Six important downstream integrin genes—Cfl1, Cfl2, Gsn, Pfn1, Pfn2, and Pfn3—in the regulation of actin cytoskeleton pathway [31, 43] were listed in Table 7. Among them, five genes were down-regulated in the chitosan group; whereas two genes were down-regulated yet one gene was up-regulated in the collagen/chitosan group. There are more down-regulated genes in the chitosan group than those in the collagen/chitosan group.

**Table 7.** Gene expression of important downstream integrin genes in the regulation of actin cytoskeleton pathway (red ⋆)

Gene symbol	Chitosan film	Collagen/chitosan film
Cfl1	0.022 ± 0.002↓	0.306 ± 0.004↓**
Cfl2	0.028 ± 0.009↓	
Gsn	0.427 ± 0.005↓	
Pfn1	0.053 ± 0.000↓	0.288 ± 0.003↓**
Pfn2	0.285 ± 0.002↓	
Pfn3		1.973 ± 0.141↑

** *P* < 0.01, fold change of one gene between chitosan and collagen/chitosan groups was considered to have a very significant difference.

Cofilin (CFL) binding to monomeric G-actin and filamentous F-actin in cells speeds up actin polymerization via severing actins and creating more positive ends, which promotes reorganizing actin filaments in the early stages of myofibril assembly and thereby regulates actin dynamics in the cytoskeleton [44]. CFL genes have two subtypes, Cfl1 and Cfl2. Gelsolin (GSN) is an actin-binding protein and is one of the most potent actin-severing proteins. The roles of GSN have been demonstrated in the regulation of cell motility [45], in the signal transduction into dynamic rearrangements of cytoskeletal architecture [46] and in the inhibition of apoptosis by stabilizing the mitochondria [47]. Profilin (PFN) is also a pivotal actin-binding protein relevant to actin polymerization and stabilization, and thus cytoskeleton maintenance [43]. PFN1, PFN2 and PFN3 are three isoforms of PFN. PFN1 regulates actin polymerization and cytoskeletal reorganization. The down-regulation of PFN2 significantly reduces cell migration and invasion of esophageal cancer cells (Eca109 cells) [48]. PFN3 is an important regulator in cell elongation and F-actin organization [49]. In the chitosan group, five down-regulated genes (Cfl1, Cfl2, Gsn, Pfn1 and Pfn2) suggested that the chitosan films might cause defects in actin-binding proteins in the PC12 cells and thus lead to abnormal actin cytoskeleton and a reduction in anti-apoptotic ability of cells. Instead, in the collagen/chitosan group, two down-regulated genes (Cfl1 and Pfn1) and one up-regulated gene (Pfn3) implied a weaker impact of collagen/chitosan films on actin cytoskeletal dynamics in the PC12 cells.

To sum up, the adsorbed proteins (F2 and FN1) on the two materials were able to influence the regulation of actin cytoskeleton pathway by regulating the expression of F2r and integrin genes in the cells. Compared to the collagen/chitosan films, the chitosan films enabled the down-regulation of five important downstream integrin genes (Cfl1, Cfl2, Gsn, Pfn1 and Pfn2) in the PC12 cells. This led to abnormal cytoskeletal structure and a reduction in the ability to suppress apoptosis, thereby indicating the inhibitory effect of chitosan films on cell adhesion and growth.

**Figure 6.**
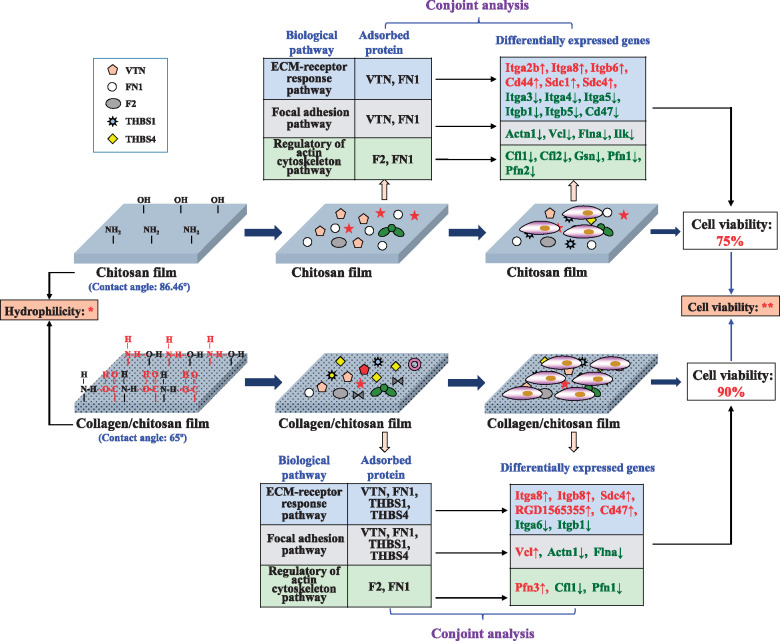
The different molecular mechanisms of PC12 cell growth affected by the chitosan films and the collagen/chitosan films

This joint analysis combined the transcriptomic analysis with the proteomic profiles and revealed the different molecular mechanisms influenced by the chitosan films and by the collagen/chitosan films. The adsorbed proteins (VTN, FN1, F2, THBS1 and THBS4) on the two materials regulated the expression of the receptor genes which interact with them mainly in three aspects as follows (Fig. 6), to influence cell adhesion and growth.

1. In the ECM–receptor interaction pathway, compared to the collagen/chitosan group, the chitosan group had more receptor genes engaged in and showed higher expressions of differentially expressed genes for the receptors binding to the adsorbed proteins. This indicated that the greater impact of chitosan films on the ECM–receptor reaction pathway was attributed to the expression of integrin-, proteoglycan-, glycoprotein- and Ig superfamily-encoding genes regulated by the adsorbed proteins on the surface.
2. The adsorbed proteins on two material surfaces influenced the focal adhesion pathway by regulating the expression of integrin genes in cells. The chitosan group had a larger number of down-regulated genes and greater down-regulation compared to those in the collagen/chitosan group. The chitosan films had a stronger inhibitory effect on PC12 cell adhesion and growth.
3. The adsorbed proteins (F2 and FN1) on the two material surfaces were able to influence the regulation of actin cytoskeleton pathway by regulating the expression of F2r and integrin genes in the cells. By enabling the down-regulation of five important downstream integrin genes and further causing abnormal cytoskeletal structure and a reduction in the ability to suppress apoptosis, the chitosan films offered a greater impact on cell adhesion and growth.

## Conclusion

This paper concluded that the difference in hydrophilicity between the chitosan and the collagen/chitosan films (collagen/chitosan films was more hydrophilicity) contributed to different types and amounts of adsorbed proteins (more FN1, THBS1, THBS4 and F2 were absorbed on the collagen/chitosan surface). The adsorbed proteins functioned differently to regulate the expressions of important downstream genes in three biological pathways (ECM-receptor interaction pathway, focal adhesion pathway, regulation of actin cytoskeleton pathway), ultimately resulting in the significantly difference in cell viability on two material surfaces (cell viability was higher on the collagen/chitosan films than that on the chitosan films). More importantly, this study opened up new lines of inquiry on how to holistically investigate the interactions among material surface properties, adsorbed protein and gene expression relevant to cell adhesion and growth. This established technical route is able to applied for any other biomaterials to reveal the molecular mechanisms of interactions among biomaterial, proteins and cell behaviors.

## Supplementary data

Supplementary data are available at *REGBIO* online.

## Supplementary Material

rbaa030_supplementary_data
