# Supplementary material for: Integrated transcriptomic and proteomic study on the different molecular mechanisms of PC12 cell growth on chitosan and collagen/chitosan films
Source: Regen Biomater. 2020 Aug 31;7(6):553–65. doi: 10.1093/rb/rbaa030 (PMC7748450; doi:10.1093/rb/rbaa030)
Supplement: rbaa030_supplementary_data [file rbaa030_supplementary_data.docx]

**Supporting Information**

**Title:** Integrated Transcriptomic and Proteomic Study on the Different Molecular Mechanisms of PC12 Cell Growth Affected by Chitosan Films and Collagen/chitosan Films

**Author:** Xiaoying Lü*, Yan Huang, Yayun Qu, Yiwen Zhang, Zequn Zhang

**Supplementary Table 1:** Number of the differentially expressed genes and the related biological pathways of cell adhesion and growth in the PC12 cells cultured on two material surface.

| No. | Pathway | Chitosan film | Collagen/chitosan film |
| --- | --- | --- | --- |
| 1 | PI3K-Akt signaling pathway | 187 | 106 |
| 2 | MAPK signaling pathway | 139 | 70 |
| 3 | Ras signaling pathway | 128 | 61 |
| 4 | Focal adhesion | 126 | 72 |
| 5 | Regulation of actin cytoskeleton | 118 | 64 |
| 6 | Rap1 signaling pathway | 108 | 60 |
| 7 | cAMP signaling pathway | 100 | 56 |
| 8 | Chemokine signaling pathway | 95 | 53 |
| 9 | Hippo signaling pathway | 89 | 46 |
| 10 | cGMP-PKG signaling pathway | 82 | 44 |
| 11 | Insulin signaling pathway | 81 | 47 |
| 12 | Cell cycle | 80 | 46 |
| 13 | AMPK signaling pathway | 78 | 51 |
| 14 | Oxytocin signaling pathway | 77 | 34 |
| 15 | FoxO signaling pathway | 76 | 46 |
| 16 | Neurotrophin signaling pathway | 76 | 37 |
| 17 | Sphingolipid signaling pathway | 74 | 35 |
| 18 | Jak-STAT signaling pathway | 70 | 30 |
| 19 | Thyroid hormone signaling pathway | 65 | 40 |
| 20 | TNF signaling pathway | 62 | 41 |
| 21 | T cell receptor signaling pathway | 62 | 24 |
| 22 | Estrogen signaling pathway | 58 | 26 |
| 23 | ErbB signaling pathway | 56 | 22 |
| 24 | HIF-1 signaling pathway | 55 | 33 |
| 25 | Fc gamma R-mediated phagocytosis | 52 | 28 |
| 26 | ECM-receptor interaction | 51 | 29 |
| 27 | TGF-beta signaling pathway | 48 | 22 |
| 28 | Tight junction | 47 | 25 |
| 29 | NF-kappa B signaling pathway | 46 | 25 |
| 30 | p53 signaling pathway | 44 | 28 |
| 31 | Gap junction | 44 | 22 |
| 32 | B cell receptor signaling pathway | 40 | 20 |
| 33 | Adherens junction | 39 | 23 |
| 34 | RIG-I-like receptor signaling pathway | 37 | 15 |
| 35 | Apoptosis | 36 | 18 |
| 36 | VEGF signaling pathway | 34 | 15 |
| 37 | mTOR signaling pathway | 31 | 21 |
| 38 | Cytosolic DNA-sensing pathway | 30 | 16 |
| 39 | Wnt signaling pathway | 78 |  |
| 40 | Prolactin signaling pathway | 43 |  |
| 41 | Calcium signaling pathway |  | 51 |
| 42 | PPAR signaling pathway |  | 21 |
